# Endodontic Management of an Infected Primary Molar in a Child with Agenesis of the Permanent Premolar 

**DOI:** 10.22037/iej.2017.25

**Published:** 2017

**Authors:** Saeed Asgary, Mahta Fazlyab

**Affiliations:** a * Iranian Center For Endodontic Research, Research Institute of Dental Sciences, Dental School, Shahid Beheshti University of Medical Sciences, Tehran, Iran;*; b *Department of Endodontic, Dental Branch, Islamic Azad University, Tehran, Iran*

**Keywords:** Calcium-Enriched Mixture, CEM cement, Endodontic, Primary Molar, Tooth Agenesis, Tooth Missing

## Abstract

Missing of mandibular second premolar is one of the most common types of tooth agenesis. In such cases, maintenance of the primary second molar, if possible at all, can prevent many treatment procedures in future. The present case report represents the endodontic management of a necrotic left mandibular primary second molar that had developed an abscess. Considering the missing of the permanent successor, the tooth was disinfected during endodontic preparation and the root canal system was filled with calcium-enriched mixture (CEM) cement in the same session. After 12 months of regular follow-up, not only the tooth was functional and symptom-free, but also healing of the inter-radicular bone lesion and re-establishment of the lamina dura was indicative of treatment success. Further trials are suggested to confirm CEM biomaterial use for management of infected primary molars associated with endodontic lesion.

## Introduction

Hypodontia, missing of one to six teeth, is one of the most common dental developmental anomalies in human [1]. A systematic review reported the overall prevalence of 6.4% for this phenomenon . Hypodontia is found more frequently in females than males [2]. Second premolars are the most common congenitally missing teeth [3]. In the majority of cases, the etiology is genetic with autosomal dominant inheritance mode [4]. Also chemotherapy or radiotherapy, trauma, medicines or infections such as osteomyelitis and rubella, can affect the proliferation of the tooth bud cells [4, 5].

For management of infected primary molars, in cases with agenesis, selection of a beneficial treatment plan with the best results over the long term brings up a challenge ahead of the clinician. Two different approaches are possible. *First*, extraction of the primary second molar (PSM) to enable the mesial drifting of the permanent first molar [6] which is indicated in cases of pulpal pathology, large restoration, carious lesions close to the pulp, normal or pathologic root resorption or orthodontic reasons naming crowding of the permanent dentition, differences in tooth sizes between deciduous and permanent teeth [3]. The *second* approach is to maintain the PSM as long as possible to avoid the potential complexity of inclination-free space closure and possibility of future periodontal issues [7]. The deciduous tooth is often retained beyond the time of normal exfoliation, meaning an extended life for that tooth [8]; most retained PSM can be maintained until adult age. In this way, the retained PSM acts as a space maintainer and a semi-permanent solution [9]. It may also prevent resorption of the alveolar bone [8]. The overall prognosis of primary teeth kept *in situ* is favorable [6]. Studies have provided evidence that retaining PSM in a younger patient can be considered a reasonable treatment option [8].

**Figure 1:**
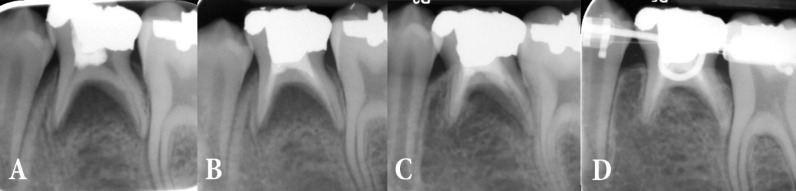
*A)* Pre-operative radiograph of the mandibular second primary molar with previous pulpotomy; note the missing of permanent second premolar; *B)* Immediate post-endodontic treatment radiograph of the tooth and root filling with CEM cement; *C)* Six-month follow-up radiography; *D)* After 12 months the tooth is asymptomatic and fully functional; note the complete inter-radicular bone healing

When extensive caries affects the dental pulp of primary molars, vital pulp therapy becomes necessary; however, if pulpal inflammation and infection extends to the root canal, then a pulpectomy procedure is indicated [10]. The treatment procedure, similar to permanent dentition, involves root canal preparation and obturation with gutta-percha; however, dissimilar to permanent dentition, the root canal system of primary molars, present enormous variations and irregularities. Best current evidence, approves mineral trioxide aggregate (MTA) as the root canal sealing/filling biomaterials for endodontic treatment of primary second molars without successors [11]. Calcium-enriched mixture (CEM) cement as an endodontic biomaterial has many properties that makes it suitable for such treatments. The main components of CEM cement are different from MTA [12]. CEM cement offers favorable physical properties such as flow, film thickness, and primary setting time [13]. The sealing ability of CEM is similar to MTA [14].Whilst the antibacterial properties of CEM cement is better than MTA and almost similar to calcium hydroxide [15], the antifungal properties of CEM on *Candida albicans* has been proved to have close proximity to MTA; they both induce complete death of fungal cells after 24 h [16]. The alkaline pH of CEM cement (~11) plays an important role in antimicrobial properties of this biomaterial [13, 15].

This report represents the one-year successful treatment outcome of a retained necrotic PSM without permanent successor that had developed an endodontic abscess in the inter-radicular area. The tooth chamber and root canals were sealed/filled with CEM cement.

## Case Report

A 12-year old girl came to a private dental clinic complaining of pain and discomfort in the left mandibular area. Her parents did not mention any contributory medical issue. Upon clinical examination, a localized swelling in the PSM area was detected that was tender on palpation. The PSM had a large amalgam filling. A parallel diagnostic radiography was representative of a previous pulpotomy treatment that according to the patient’s parents was done almost 2 years before. The noteworthy issue was the missing of the permanent second premolar. Inter-radicular lucency extending to the periradicular area was indicative of the treatment failure (Figure 1A). 

Tooth extraction carried the risk of mesial drifting of the permanent first molar. The situation was discussed with patient’s parents and the final decision was made. Treatment plan included endodontic treatment of the necrotic tooth. Should the problem continue, the alternative approach would be tooth extraction. 

After administration of local anesthesia through infiltration of lidocaine with 1:80000 epinephrine, a small incision was made on the most prominent surface of the localized swelling to enable drainage of the puss. Then the amalgam filling was removed with a high speed handpiece. The underlying zinc-oxide eugenol was also removed that allowed drainage through the canals. Four root canals (distobuccal/lingual and mesiobuccal/lingual) were detected. The root canals were irrigated with 5.25% NaOCl complemented by slight agitation of the solution with #20 and 30 H-files (Dentsply Maillefer, Ballaigues, Switzerland). This process continued for almost 15 min and the irrigation solution was refreshed every 5 min. Then the canals were dried with paper points (AriaDent, Asia Chemi Teb. Co., Tehran, Iran).

CEM cement liquid and powder (BioniqueDent, Tehran, Iran) were mixed according to the manufacturer’s instructions. The paste was carried into the canals, using a carrier gun and packed with hand pluggers (Dentsply Maillefer, Ballaigues, Switzerland) and paper points. Then the tooth was restored with amalgam in the very same session. After radiographic checking (Figure 1B), chlorhexidine mouthwash was prescribed and the patient was dismissed. Because of the localization of the swelling and adequate intra/extra canal drainage, systemic antibiotic was not prescribed.

The patient was put on a regular scheduled follow-up. Her pain totally vanished the day after. After six months, the tooth was asymptomatic and radiographically checked (Figure 1C). The formation of the lamina dura confirmed treatment success. After 12 months, the tooth was in complete function and the inter-radicular bone healing had occurred (Figure 1D). In fact the tooth was so much in function and none ankylosed that it did not interfere with orthodontic treatment.

## Discussion

This report discussed the successful endodontic treatment of a necrotic PSM associated with an apical abscess by sealing/filling the root canals and pulp chamber floor with CEM cement. After one year, the tooth was functional and the inter-radicular bone lesion had healed.

In the presented case, the perfect sealing/filling of the chamber floor and root canal walls by CEM cement was a leading factor in healing of the endodontic lesion. CEM cement is a differently-formulated biomaterial that offers the combination of superior biocompatibility of MTA with appropriate setting time (<1 h), profound handling and reasonable price [13, 17-19]. CEM cement is able to produce hydroxyapatite with endogenous and exogenous ion sources [18]; the mixed cement comprises of water-soluble calcium and phosphate and immediately forms hydroxyapatite during and after setting which can cause the superior sealing ability of CEM cement. Perfect sealing of this cement can also stem from its small particle sizes [20]. Another sealing mechanism can be the calcium sulfate and calcium silicate content of the cement that may cause a slight expansion following continuous hydration after initial setting [21]. The hydroxyl ions that are formed during further crystalline maturation in aqueous environment raise the local pH that optimizes the environment for formation of more hydroxyapatite crystals [18]. Also it should not be overlooked that although the setting expansion of MTA and CEM cement are similar, the flow and film thickness of the latter cement is reported to be significantly superior and this can justify the better handling properties as well as its sealing ability [13, 22].

In addition, it is noteworthy that treatment of the present case was finished during a single visit without prescription of systemic/local antibiotics. The antimicrobial activity of the root canal filling material played an undeniable role in healing of the apical abscess. The lethal effect of calcium hydroxide and CEM cement on isolated and mixed strains of *Pseudomonas aeruginosa*, *Enterococcus faecalis*, *Staphylococcus aureus* and *Escherichia* *coli *were almost similar and both superior to that of MTA [15]. Another study has reported the antifungal activity of MTA and CEM cement against *Candida albicans* [16]. CEM cement is composed of earth metal oxide and hydroxides such as calcium oxide and calcium hydroxide, calcium phosphate, and calcium silicate. In the medium, the dissociation of calcium hydroxide particles to hydroxyl ions and elevated pH can justify the antimicrobial activity of the cement [23]. What makes this alkalinizing mechanism different from that of MTA is the better diffusion properties due to smaller particle sizes of CEM cement [20]. 

In cases of mandibular second premolar agenesis, the PSM may be left *in situ* or extracted. In some cases, the contralateral premolar and the maxillary premolars are also extracted, with spontaneous space closure or closure with orthodontic appliances. Other options are implant-supported prosthetic replacement or a tooth-supported bridge and pontic [6]. Long-term follow-ups after extraction of the PSM revealed that in most of the cases the space was closed by mesial drift and tipping of the first molar and distal drift and tipping of the first premolar, leaving a mean residual space of 2 mm [7]. However, in subjects with no crowding, with a pronounced deep bite and a hypodivergent vertical skeletal pattern, or with mandibular retrusion or generalized spacing of teeth, extraction of the SPM is contraindicated because of the difficulty in space closure without detrimental effects on facial profile [6]. In these circumstances, maintaining the PSM would be a valuable option [6, 9].

Although extraction of the PSM with conditions similar to this case seems to be more prescribed, it cannot be denied that by providing chances of abscess healing and tooth functioning, the clinician has prevented the future complexity of space maintaining. In other words, the PSM can now play a role as a space maintainer until is natural exfoliation, which is postponed due to agenesis of the permanent successor [8], or implant placement after growth ceasing.

## Conclusion

In many cases with missing of the permanent teeth, the necrotic primary tooth can be saved via endodontic management to play its role as a space maintainer. CEM cement has proven sealing, antibacterial/fungal and regenerative properties that make it a suitable biomaterial for root canal filling of the infected primary teeth.
